# Selumetinib side effects in children treated for plexiform neurofibromas: first case reports of peripheral edema and hair color change

**DOI:** 10.1186/s12887-021-02530-5

**Published:** 2021-02-06

**Authors:** Francesco Baldo, Andrea Magnolato, Egidio Barbi, Irene Bruno

**Affiliations:** 1 University of Trieste, Trieste, Italy; 2 Department of Pediatrics, Institute for Maternal and Child Health Burlo Garofolo, Trieste, Italy

**Keywords:** Selumetinib, neurofibromatosis type I, Peripheral edema, Hair color change, Case report

## Abstract

**Background:**

Plexiform neurofibromas (PNs) are congenital tumors that affect around 50 % of the subjects with neurofibromatosis type 1. Despite being histologically benign, PNs can grow rapidly, especially in the pediatric age, and cause severe morbidities. In the past, various therapeutic approaches have been proposed to treat these masses, none of which obtained valuable results. Selumetinib, an inhibitor of mitogen-activated protein kinase (MEK) 1 and 2, has been the first molecule to demonstrate the ability of tackling the growth of PNs. The drug’s most common side effects, which usually are mild or moderate, include gastrointestinal symptoms (diarrhea, abdominal pain), dermatologic manifestations (maculo-papular and acneiform rash, paronychia, mucositis), and various laboratory test abnormalities (elevation of creatine kinase and aminotransferase).

**Cases presentation:**

We report two previously undescribed adverse events in pediatric patients: peripheral edema and hair color change. The first case of peripheral edema occurred in a 7-year-old boy affected by a severe form of NF1, after two years of treatment with selumetinib at the standard dose (25 mg/m^2^twice a day). The edema involved the right leg, and the patient did not complain of pain. The second case of peripheral edema occurred in a 12-year-old girl after six months of therapy with selumetinib at the standard dose, involving her lower left leg. The patient initially complained of pain in that area, but it gradually and spontaneously resolved. In both patients, all the radiological exams, including lymphoscintigraphy, pelvic and abdominal ultrasound, and doppler ultrasound of the affected limb, as well as blood tests, revealed no abnormalities. Hair color change appeared in a 4-year-old boy after six months of therapy at the standard dose. The boy’s hair, whose natural color was dark blonde, became lighter in some areas. Despite the appearance of these side effects, all the patients and their families decided to continue the treatment with selumetinib, in considerations of its clinical benefits.

**Conclusions:**

Since the use of selumetinib to treat plexiform neurofibromas is increasing in the pediatric population, clinicians should be aware of its side effects, so to decide whether continuing the treatment, reducing the dose or even interrupting it, when appropriate.

## Background

Neurofibromatosis type 1 (NF1) is a common genetic disorder that affects around 1/3000 subjects. This condition is characterized by the presence of pigmented lesions (café au lait spots, axillary, inguinal freckling, Lisch nodules of the iris, choroidal freckling), multiple cutaneous neurofibromas, brain tumors (optic nerve and central nervous system glioma), and peripheral nerve tumors (plexiform neurofibromas and malignant peripheral nerve sheath tumors). Other features include skeletal abnormalities, such as tibial dysplasia and scoliosis, renovascular hypertension, and learning disabilities.

Plexiform neurofibromas (PNs) affect around 50 % of the individuals with NF1 at some point in their life. Being congenital, PNs tend to appear in the pediatric age, reaching their peak of growth during adolescence. Although histologically benign, these tumors can generate severe morbidities, including pain, functional limitations, neurological deficits, disfigurement, and internal organs’ compression. Various medical approaches have been proposed in the past, including interferon alfa2β and imatinib, without tangible results. Complete surgical resection is often unattainable, and the persistence of tumor remnants can result in the regrowth of the mass.

Selumetinib is an ATP-independent inhibitor of mitogen-activated protein kinase (MEK or MAPK/ERK kinase) 1 and 2, which are key mediators of the RAS/RAF/MEK/ERK pathway, that is upregulated in NF-1. The drug has already been used in adult oncology in different protocols, such as in non-small cell lung cell carcinoma and uveal melanoma, with positive results. Selumenitib showed the capacity to tackle plexiform neurofibromas’ growth in pediatric patients, obtaining a reduction of the original size of the mass of at least 20 % in most of the patients [1–4]. A decrease in tumor-related pain, disfigurement, and functional impairment was also described. In consideration of these results, on April 10, 2020, selumetinib has been approved by the Food and Drug Administration (FDA) for the treatment of inoperable plexiform neurofibromas. Furthermore, it has also demonstrated a valuable, although preliminary role, in the treatment of low-grade glioma, so to represent a promising alternative to standard chemotherapy [5].

The drug’s recommended maximum posology is currently 25 mg/m^2^ twice a day, approximately 60 % of the standard adult dosage. In terms of safety profile and pharmacological toxicity, the drug is usually well tolerated. The most common side effects are mild or moderate, and they include gastrointestinal symptoms (diarrhea, abdominal pain), dermatologic manifestations (maculo-papular and acneiform rash, paronychia, mucositis), and various lab test anomalies (elevation of creatine kinase and aminotransferase elevation, neutropenia). Potentially serious adverse events of the drug, which have been observed in adult patients receiving selumetinib in combination with other chemotherapeutical agents, are rare and include ocular (retinal detachment), pneumological (pulmonary fibrosis) and cardiological manifestations (asymptomatic and reversible decrease of the left ventricular ejection fraction).

This paper reports two previously undescribed adverse effects in pediatric patients: peripheral edema and hair color change.

## Cases presentation

The first case of peripheral edema occurred to a 7-year-old boy affected by a severe form of NF1, characterized by multiple inoperable plexiform neurofibromas in the neck, thorax, dorsum, and right leg. The masses caused compression of numerous organs and nerves, resulting in dysphagia, dyspnea, and chronic pain with inadequate response to analgesic medications. The boy also presented severe renovascular hypertension that led to two episodes of stroke, from which he gradually recovered. Based on his severe symptoms and poor quality of life, at the age of 5 selumetinib was started, with a significant shrinkage of the PNs after six months of therapy, and a remarkable and constant improvement in performance status. The maximum drug dosage was 25 mg/m^2^ twice a day. After two years of treatment, in approximately three months, the boy slowly developed a unilateral swelling of the right leg. He did not complain of symptoms originating from the edematous area, nor developed any skin complications. Clinically, no other new abnormalities were found. For example, there was no evidence of palpable abdominal masses, and lymphadenopathy was not found. Ultrasonography demonstrated that the PN was in stable remission, and a doppler study showed no signs of deep venous thrombosis. Lymphoscintigraphy was normal as well. No masses were found on abdominal and pelvic ultrasonography. Routine blood laboratory tests did not reveal abnormalities. An echocardiogram also appeared normal. Therefore, since all possible causes of edema were excluded, the right leg’s swelling was attributed to the drug itself. In consideration of the absence of symptoms and the drugs’ remarkable benefits, we decided to continue the treatment with selumetinib, which is still daily taken by the patient. Peripheral edema is still present, but it has remained stable through the following months.

**Fig. 1:**
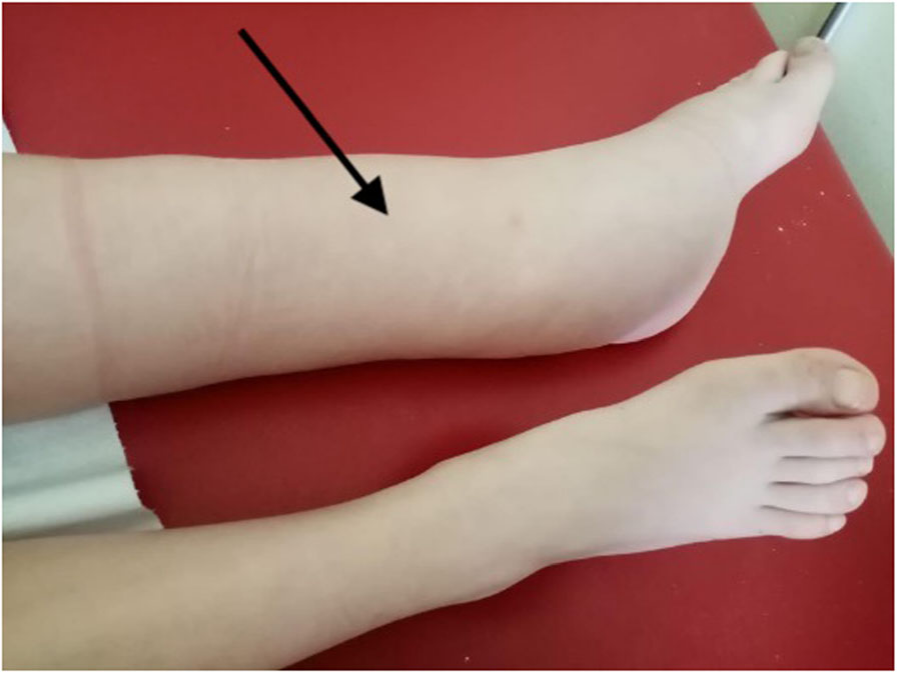
Lower left leg edema after the treatment with selumetinib

The second case of peripheral edema occurred to a 12-year-old girl that was diagnosed with NF-1 at birth. At the age of two, she underwent an MRI, which detected multiple plexiform neurofibromas in her left leg. Due to her health condition’s severity, treatment with imatinib and eventually with interferon alfa2 was started, without clinical and radiological improvement. Selumetinib was then introduced when she was 11. The maximum drug dosage was 25 mg/m^2^ twice a day. After 6 months of therapy, in which a reduction of the plexiform neurofibroma’s size was clinically and radiologically noticed, her lower left leg gradually became swollen and painful (Fig. 1). Abdominal and pelvic ultrasonography, lower limbs’ doppler ultrasound and lymphoscintigraphy, as well as echocardiogram and blood laboratory values, were all standard. The parents and the patient decided to continue the MEK inhibitor treatment as it caused a shrinkage of the original mass. Nowadays the limb’s edema is still present, but it has remained clinically stable, and the local pain gradually and spontaneously resolved. 

**Fig. 2:**
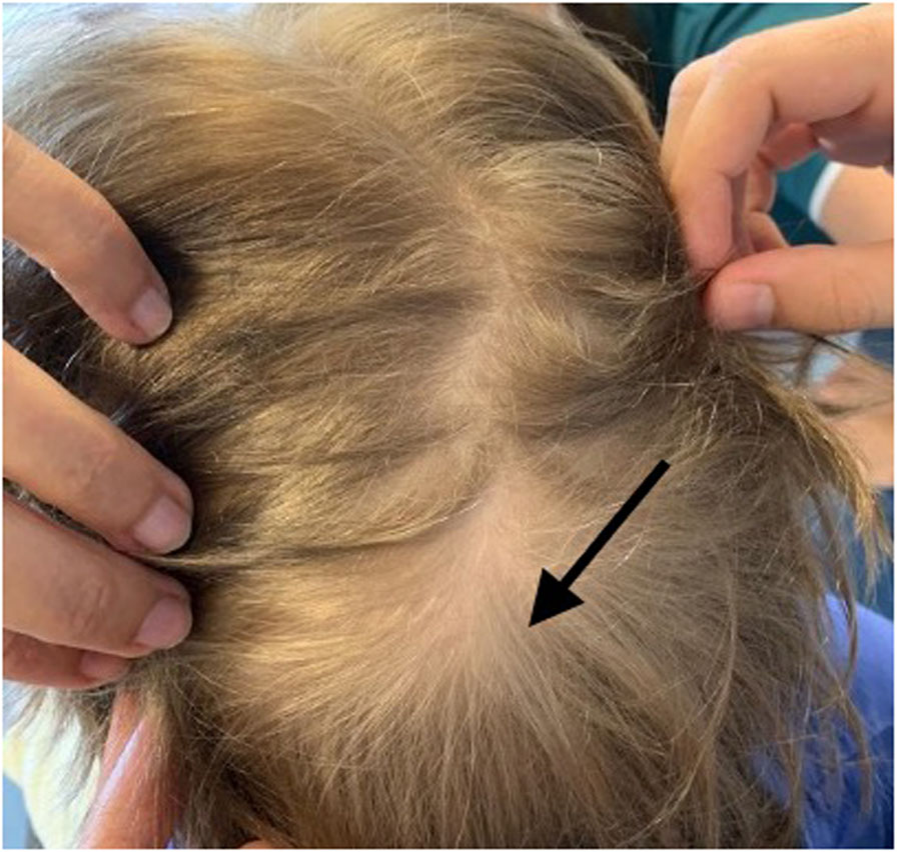
Hair color change after treatment with selumetinib

The third patient is a 4-year-old boy with multiple PNs, one of whom was surgically removed from the abdomen at one year of age. A second mass was identified during his follow up visits, located in the paravertebral region. Since the tumor appeared to be continually growing, selumetinib was started when the boy was 3-years old. Apart from some mild gastrointestinal symptoms (abdominal cramps without diarrhea), the treatment was well tolerated. After six months of therapy, the patient’s parents began to notice a discoloration of their son’s hair (Fig. 2). The boy’s hair, whose natural color was dark blonde, became light-blonde and almost white in some areas. The patient’s parents considered the symptom trivial, and the patient continued the treatment due to the good results on the mass reduction. At present the boy still has some patch of hair lighter than his original color.

## Discussion and conclusion

This is the first case report of pediatric subjects developing peripheral edema and hair color change while treated with selumetinib.

Selumetinib, like other MEK inhibitors, has already been associated with the onset of peripheral edema in adult patients. A 2017 metanalysis evaluated the incidence of peripheral edema in patients with cancer treated with MEK inhibitors [6]. Among all the studies included in the paper, peripheral edema was reported in 13 RCTs and appeared in a total of 284 patients, with an incidence ranging from 11–47.8 % in the various cohorts examined. The degree of swelling was usually mild or moderate, and it didn’t interfere with the prosecution of the treatment. High-grade peripheral edema, possibly requiring a dose reduction or even the suspension of the drug, was reported in 8 studies, with an incidence that ranged from 0 to 4.3 %. As for the timing of appearance of the symptom in adult patients, no conclusive data are available at the moment. Peripheral edema was more common in patients treated with selumetinib, rather than with trametinib or other MEK inhibitors, and it has not been influenced by the presence of other concurrent medical therapies. Apart from the involvement of lower extremities, facial edema has also been reported [7]. The mechanism through which selumetinib causes this clinical feature is not well understood. Peripheral edema is a complex process that involves numerous variables, including hydrostatic and oncotic forces, and vascular permeability. However, none of these elements, alone or combined, appears sufficient, at the moment, to explain the pathophysiology of this process. For example, none of the patients whose cases were described above presented heart, kidney or liver failure, nor a deep vein thrombosis or an ongoing malignant process. Furthermore, no significant abnormalities were found on laboratory tests.

Hair color change was never reported in pediatric patients treated with selumetinib in the past [8]. Systemic drugs are often associated with changes in hair’s quantity and quality, such as alopecia and hypertrichosis, while they rarely modify their color (i.e. depigmentation and repigmentation). The mechanism of hair depigmentation in patients treated with selumetinib is only partially understood. The MAPK pathway is utilized by physiologic c-KIT-MITF signaling in promoting pigment production [9]. In adults, hair depigmentation, like all the other dermatological side effects of selumetinib, is completely reversible once treatment is discontinued [10]. The application of hair dye might be considered in order to restore the patient’s original color.

In light of selumetinib’s positive results in reducing plexiform neurofibromas’ growth, it can be assumed that this drug will be used more frequently in the near future. Physicians should be aware of all its possible adverse events and how to deal with them. Awareness that peripheral edema and hair color change are uncommon, yet not disturbing, manifestations, may help clinicians opt for the continuation of the treatment, rather than a dose reduction or even the drug interruption, allowing for growth control of plexiform neurofibromas. However, further studies are needed to determine the frequency of these side effects in longer follow-up protocols, to understand their origin and to find an appropriate treatment.

## Data Availability

Not applicable.

## Abbreviations

NF1: Neurofibromatosis type 1
PN: Plexiform neurofibroma
MEK: Mitogen-activated protein kinase
ERK: Extracellular-signal-regulated
RAF: Rapid accelerated fibrosarcoma
FDA: Food and Drug Administration
MRI: Magnetic resonance imaging
MIFT: Melanocyte inducing transcription factor
